# Which Subspecialties Do Female Orthopaedic Surgeons Choose and Why? Identifying the Role of Mentorship and Additional Factors in Subspecialty Choice

**DOI:** 10.5435/JAAOSGlobal-D-19-00140

**Published:** 2020-01-20

**Authors:** Rachel A. Bratescu, Stephanie S. Gardner, Jaclyn M. Jones, Todd E. Siff, Bradley S. Lambert, Joshua D. Harris, Shari R. Liberman

**Affiliations:** From the Houston Methodist Hospital Orthopedics & Sports Medicine, Outpatient Center, Houston, TX

## Abstract

Limited data exist delineating the reasons women choose subspecialties within orthopaedics.

**Purpose:**

(1) To perform a survey that determines subspecialties female orthopaedic surgeons select and (2) to analyze the motivations behind their choices.

**Methods:**

A 10-question survey was distributed via e-mail to the Ruth Jackson Orthopaedic Society (RJOS), Texas Orthopaedic Association (TOA), and to a private internet page for women in Orthopaedics, which covered the area of subspecialty practice, motivations, and demographic data. Practicing female orthopaedic surgeons, fellows, or fellowship-matched residents were included. Respondents' ranked motivations when deciding for or against a subspecialty were analyzed and comparisons made.

**Results:**

Of the 304 survey responses, 288 met inclusion criteria. The most common subspecialties were hand (24.0%), pediatrics (22.6%), and sports medicine (16.3%). A higher proportion of younger surgeons are electing to subspecialize in sports medicine, whereas a lower proportion of younger surgeons are pursuing general orthopaedics. Top-ranked reasons for selecting a subspecialty were personal satisfaction (50.8%), intellectual stimulation (42.1%), and strong mentorship (37.4%). The most common reason for not selecting a subspecialty was lack of interest (60.6%).

**Conclusion:**

Strong mentorship was the largest extrinsic/modifiable factor that affected the decision-making process. A continued focus on mentorship will be necessary to encourage future female orthopaedic surgeons to enter this field and inspire them to explore a different set of subspecialties.

The percentage of women attending medical school has been steadily increasing in recent years. In 2017, 51% of medical students were women.^1^ Orthopaedic surgery, when compared with other surgical subspecialties, still has the lowest percentage of women residents.^2^ Despite this, the number of women entering the field of orthopaedics has been increasing. In 2016, women made up only 6.5% of orthopaedic surgeons within the AAOS membership and 15% of the current orthopaedic surgery residents.^3,4^

Previous studies have explored why women pursue orthopaedic surgery residency, but limited data exist supporting the reasons why women choose different orthopaedic fellowships and subsequent subspecialties.^5-7^ According to the 2010 to 2014 fellowship match data for orthopaedic surgery, pediatric orthopaedic surgery had the highest percentage of female applicants (25%), followed by foot and ankle (14%).^8^ A 2016 study of residents, fellows, practicing, and retired surgeons asked why women chose orthopaedic surgery, and they found the most common reasons to be enjoyment of manual tasks, followed by professional satisfaction and intellectual stimulation.^9^ The study also showed that among participants a higher proportion chose hand (22%), general orthopaedics (19%), and pediatric orthopaedic surgery (16%) as subspecialties.^9^ These trends highlight the need for further investigation into the reasons women are drawn to these particular subspecialties.

The purpose of this study was to determine which (1) subspecialties female orthopaedic surgeons select and (2) analyze the motivations behind their choices. We hypothesized that most female orthopaedic surgeons continue to choose the field of pediatric orthopaedic surgery, and their primary motivation for doing so is related to mentorship opportunities.

## Methods

A 10-question custom survey was created in January 2018, with content based on practice demographics and the reasons why participants chose their practicing subspecialty (Supplemental Document, http://links.lww.com/JG9/A61). The survey was created, reviewed, and agreed on by three of the authors (R.A.B., S.S.G., and S.R.L.). The senior author (S.R.L.) made the final decision regarding any disagreement on structure or content. Specific subspecialties included were the same as those available in the American Academy of Orthopaedic Surgeons (AAOS) 2016 census (Table 1). Participants were given the ability to choose more than one subspecialty. This survey was distributed via e-mail to all active members of the Ruth Jackson Orthopaedic Society (RJOS), Texas Orthopaedic Association (TOA), and a Facebook group for women in orthopaedics from January to March 2018. This private internet group included retired and practicing orthopaedic surgeons as well as residents and fellows. Physicians included in this study were required to be practicing female orthopaedic surgeons, fellows, or residents who were fellowship-matched (Table 2). Exclusion criteria included incomplete response, participants who were not fellowship-matched, or participants of male sex.

**Table 1:** Orthopaedic Subspecialties

Subspecialty
Sports medicine
Total joint
Hand
Adult spine
Trauma
Pediatric
Foot/Ankle
Adult knee
Shoulder/Elbow
Arthroscopy
Adult hip
Nonsurgical practice
Orthopaedic oncology
Pediatric spine
Disability/Legal
Rehab/prosthetics/orthotics
Other
General

**Table 2:** Division of Survey Participants by Experience Level

Years in Practice	No. of Respondents	Percent
PGY 5	14	4.9
Fellow	22	7.6
<1	23	8.0
1-5	80	27.8
5-10	58	20.1
10-15	35	12.2
15-20	12	4.2
20-25	18	6.3
>25	26	9.0
Total	288	100

The survey included written consent for approval to use their information in a research study. Results were collected via SurveyMonkey. All of the respondents' first, second, and third ranked motivations when deciding for or against a subspecialty were analyzed and comparisons made.

## Results

Three hundred and five survey responses were received during the study period, of which 288 met inclusion criteria. According to the 2016 AAOS census data citing 6.5% of practicing surgeons being female, the response rate to this survey from our target audience was estimated at 15.9%.^4^ Of responses received, 39% of surveys were from the private internet group, 59.3% from RJOS, and only 1.6% from TOA.

Among the 288 respondents, the most common subspecialties for female orthopaedic surgeons were hand (24.0%), pediatrics (22.6%), and sports medicine (16.3%) (Table 3). For the surgeons who chose not to pursue a subspecialty, their reasons were family-related, interest in many subspecialties, and graduating residency at a time when most surgeons went straight into practice. The most common type of practice for female orthopaedic surgeons who responded to the survey was academic (38.9%) (Table 4). In terms of board status, 71.2% of respondents were board-certified and 25.7% were board-eligible. Ninety-two percent of respondents worked full time. Subspecialties including orthopaedic oncology, adult spine, pediatric spine, total joints, trauma, and sports medicine were found overall to possess a younger female population (>70% younger than 10 years in practice). Specifically, when analyzing specialty selection versus experience classification (or years in practice), it was found that a significantly higher proportion of recently graduated surgeons are electing to subspecialize in sports medicine, whereas a markedly lower proportion of recently graduated surgeons are electing to pursue general orthopaedics.

**Table 3:** Study Sample for Area of Subspecialty

Subspecialty	Study Sample Observations	Percent
Hand	69	24.0
Pediatric	65	22.6
Sports medicine	47	16.3
Total joint	25	8.7
Trauma	24	8.3
Foot/Ankle	24	8.3
General	22	7.7
Orthopaedic oncology	22	7.6
Pediatric spine	13	4.5
Adult spine	9	3.1
Shoulder/Elbow	7	2.4
Arthroscopy	7	2.4
Adult knee	2	0.7
Adult hip	2	0.7
Nonsurgical practice	2	0.7
Disability/Legal	0	0.0
Total study participants	288	100.0

Data are shown as number and percentage of population selecting for individual subspecialties.

**Table 4:** Survey Responses for the Type of Practice

Practice Type	Study Sample Observations	Percent
Private	79	27.4
Academic	112	38.9
Hospital	60	20.8
Military	6	2.1
Total	288	100

Data are shown as number and percentage of population for type of practice for survey respondents.

Personal satisfaction (20.7%) and intellectual stimulation (17.8%) were the most common motivations women cited across all subspecialties for pursuing a career in orthopaedic surgery. This was then followed by the pathology of patients (9.3%) and strong mentorship (8.9%). Although personal satisfaction and intellectual stimulation are intrinsic factors that affect subspecialty choice, mentorship was found to be the highest ranked extrinsic factor that influenced subspecialty choice. Between subspecialties, the motivations for choosing a particular subspecialty varied. Those specializing in hand surgery cited enjoyment of small detailed procedures (28.9%), whereas for sports medicine it was personal history/interest in sports (44.7%). The top reasons listed for not choosing a different orthopaedic subspecialty was a lack of interest in other subspecialties (60.6%), followed by other (11.9%) and a lack of strong mentorship (7.9%).

## Discussion

This study showed that female orthopaedic surgeons are choosing hand, pediatrics, and sports medicine over other subspecialties in this study population. Their top motivations in doing so include personal satisfaction and intellectual stimulation. Among the survey participants, mentorship was found to be the largest extrinsic factor affecting a respondent's decision to pursue a subspecialty, which is in accordance with our initial hypothesis.

Although the number of women in medical school has increased to over 50% of graduating classes, orthopaedic surgery is lagging behind in increasing the percentage of women in the field.^1,2^ Women make up only 6.5% of orthopaedic surgeons in America according to the AAOS census published in 2016.^4^ Previous studies have examined why women pursue orthopaedic surgery and found that the lack of awareness and mentorship have discouraged women from choosing this field.^2,6^ Strategies have been implemented to increase interest including societies such as the Ruth Jackson Orthopaedic Society (RJOS) and the J. Robert Gladden Society, which have workshops dedicated to medical students.^2^ Our goal was to evaluate which subspecialties women pursue and the motivations behind their choices, in hope of identifying ways to increase sex diversity within the orthopaedic surgery subspecialties.

Historically, women have pursued hand and pediatric orthopaedic surgery over other subspecialties.^9^ Our study showed that as a group they continue to choose these subspecialties more often than others. We think this pervasive trend in subspecialty choice has a direct correlation with more readily available mentorship opportunities. A promising observation among our survey respondents demonstrated that more recently graduated surgeons are pursuing a wider range of subspecialties than reported in the past, although an in-depth analysis of this pattern for the purposes of this study was not feasible.

Our survey showed that mentorship was the most common extrinsic motivator when deciding both for and against an orthopaedic subspecialty. Moving forward, a focus on modifiable extrinsic factors will be necessary to promote diversity within orthopaedics. In this survey, we did not explore the similarities or differences between the respondent and their mentor. It should be noted that mentors may not represent the same sex or subspecialty as the mentee. Gaining exposure to different subspecialties can help facilitate someone's interest in the chosen field, which includes time spent with a mentor outside of clinical responsibilities. We think that mentorship has a pivotal role in drawing an individual to a particular field, which has been seen in other studies.^2,9,10^ Mentorship has been found to be a factor in deciding on a career choice for women orthopaedic surgeons who are in academics, which was also seen in our study.^10^ A continued and more stringent focus on interest groups and organizations that promote not only recruiting aspiring female orthopaedic surgeons but also increasing the diversity of the subspecialties explored will be paramount moving forward. Mentorship opportunities can be found through organizations such as the Ruth Jackson Orthopaedic Society (RJOS), the American Academy of Orthopaedic Surgeons (AAOS), and The Perry Initiative. Moreover, trainees should be encouraged to seek out mentors in their field to facilitate their career aspirations. The results of this study can help educate current and future residents who are deciding on a career path and direct future research addressing sex differences or disparities in the field of orthopaedic surgery.

Limitations of our study include the limitations of an electronically distributed custom survey not using the Delphi method and the logistical challenge of reaching a broad sample of practicing female orthopaedic surgeons. Through this method it is not possible to capture all of our targeted survey population and estimate an accurate response rate. A significant percentage (38.9%) of our survey respondents described their practice type as academic, which was considerably more than the general AAOS population at 19%.^4^ Understanding that the AAOS data include both male and female respondents, we still think this represents a significant difference. For this reason, our study population may more closely represent the views of practicing female orthopaedic surgeons in an academic setting as opposed to hospital-based or private practice. Our primary goals in this study were to explore which subspecialties female orthopaedic surgeons choose and the motivations behind their choices. For this reason, we did not collect demographic data including age, ethnicity, and income status, which may potentially influence subspecialty choice and represent meaningful areas of future investigation.

## Conclusion

Our survey shows that female orthopaedic surgeons are still choosing similar subspecialties, including hand and pediatric orthopaedic surgery. Strong mentorship in a subspecialty was the largest extrinsic or modifiable factor that affected a female orthopaedic surgeon's decision-making process. A continued focus on mentorship will be necessary to both encourage future female orthopaedic surgeons to enter this field and inspire them to explore a different set of subspecialties.

## Supplementary Material

SUPPLEMENTARY MATERIAL
