# Supplementary material for: Which Subspecialties Do Female Orthopaedic Surgeons Choose and Why? Identifying the Role of Mentorship and Additional Factors in Subspecialty Choice
Source: J Am Acad Orthop Surg Glob Res Rev. 2020 Jan 20;4(1):e19.00140. doi: 10.5435/JAAOSGlobal-D-19-00140 (PMC7028786; doi:10.5435/JAAOSGlobal-D-19-00140)
Supplement: SUPPLEMENTARY MATERIAL [file jg9-4-e19.00140-s001.docx]

**Survey Questions**

*** 1. Do you consent to answer the rest of these questions that may be used for research purposes on the discrepancies of male and female orthopedic surgeons?**

Yes

No

*** 2. Do you consent to answer the rest of these questions that may be used for research purposes on the discrepancies of male and female orthopedic surgeons?**

Yes

No

*** 3. What is your subspecialty?**

Sports Medicine

Total Joint

Hand

Adult Spine

Trauma

Pediatric

Foot/Ankle

Adult Knee

Shoulder/elbow

Arthroscopy

Adult Hip

Non-operative Practice

Orthopedic Oncology

Pediatric Spine

Disability/legal ortho

Rehab/prosthetics/orthotics

Other

General. What was your reason for not pursuing a fellowship?

*** 4. What was your motivation to pick your subspecialty? Please rank the top three reasons why you picked your subspecialty.**

Enjoy working with others in your subspecialty

Enjoy small, detailed procedures

Strong mentors throughout training

Enjoy large, more open procedures

Family member(s) in the same subspecialty

Positive perception of women in your subspecialty

Other

Ability to maintain work/life balance

Pathology of patients

Compensation/financial security

Exposure to your specific subspecialty in training

Self or family member had orthopedic surgery in your subspecialty

Personally satisfying

Personal history/interest in sports

Age range of patients

Intellectually stimulating

*** 5. Why were you not interested in other subspecialties? Please rank the top three reasons why you were not interested in other subspecialties.**

Lack of interest in other subspecialties

Perception that too much strength is required for other subspecialties

Dislike the operating room

Negative perception of women in other subspecialties

Lack of exposure in training to other subspecialties

Lack of strong mentorship

Negative personal experience with surgeons in other subspecialties

Negative personal experience with other subspecialties

No sports history or interest

Fear of inability to maintain work/life balance

Lack of camaraderie in other subspecialties

Other

*** 6. How long have you been in practice?**

PGY1-4

PGY-5 or fellowship-matched

Fellow

Less than 1 year

1-5 years

5-10 years

10-15 years

15-20 years

20-25 years

Over 25 years

*** 7. Are you board certified?**

Yes

No

Board eligible

If you responded 'No', please explain why:

*** 8. Are you full time or part time?**

Full time

Part time

*** 9. What type of practice do you currently have?**

Private

Academic

Hospital

Military

Still in training

*** 10. In what geographic location are you practicing?**

Northeast

Southeast

Midwest

Southwest

West

Other location:

*** 11. What is your sex?**

Female

Male

Other
